# Posttraumatic Growth and Coping Strategies Among Patients With Head and Neck Cancer: Do Approach Coping and Avoidant Coping Predict Posttraumatic Growth Over Time?

**DOI:** 10.3389/fpsyg.2021.716674

**Published:** 2021-10-26

**Authors:** Nik Ruzyanei Nik Jaafar, Norhaliza Abd Hamid, Nur Amirah Hamdan, Rama Krsna Rajandram, Raynuha Mahadevan, Mohd Razif Mohamad Yunus, Hazli Zakaria, Mohammad Farris Iman Leong Bin Abdullah

**Affiliations:** ^1^Department of Psychiatry, Universiti Kebangsaan Malaysia Medical Centre, Cheras, Malaysia; ^2^Lifestyle Science Cluster, Advanced Medical and Dental Institute, Universiti Sains Malaysia, Penang, Malaysia; ^3^Department of Oral and Maxillofacial Surgery, Universiti Kebangsaan Malaysia Medical Centre, Cheras, Malaysia; ^4^Department of Otorhinolaryngology, Universiti Kebangsaan Malaysia Medical Centre, Cheras, Malaysia

**Keywords:** posttraumatic growth, approach coping, avoidant coping, head and neck cancer, longitudinal study, Malaysia

## Abstract

Despite an enormous number of studies addressing the importance of posttraumatic growth (PTG) among cancer patients, the literature lacks data regarding how different coping strategies affect PTG among head and neck cancer (HNC) patients over time. This longitudinal study investigated the PTG trend and coping over 5–7months among a cohort of HNC patients within the first year after their diagnosis. It determined an association between coping strategies and PTG over time. The study’s HNC respondents were administered a socio-demographic and clinical characteristics questionnaire during their baseline assessments. Additionally, the Malay versions of the “PTG Inventory-Short Form” (PTGI-SF) and the “Brief Coping Orientation to Problems Experienced Inventory” (Brief COPE) were administered during respondents’ baseline assessments and follow-up assessments (5–7months after the baseline assessments). In total, 200 respondents reported an increasing PTG trend and approach coping (active coping, planning, positive reframing, acceptance, emotional support, and instrumental support) and a decreasing trend of avoidant coping (self-distraction and denial) over time. Two approach coping strategies (acceptance and planning) significantly increased PTG while denial was the only avoidant coping strategy that significantly lowered PTG, after controlling for socio-demographic and clinical characteristics, over time. Our study’s findings identified the need to incorporate psychosocial interventions that enhance approach coping and reduce avoidant coping into HNC patients’ treatment regimes.

## Introduction

*Cancer* is generic term describing a group of diseases that feature abnormal cell growth beyond cells’ boundaries, invading adjoining tissues and spreading the abnormal cells to other organs. Cancer is a leading cause of death globally, accounting for 10 million mortalities in 2020 (World Health Organization, 2021). In Malaysia, cancer is equally common, with a 5-year prevalence of 128,018 cases and an incidence of 48,639 cases in 2020. Head and neck cancer (HNC) is the fourth most common cancer among men in Malaysia, representing 7.4% of all cancer cases in the country in 2020 (Globocan, 2020). Due to its association with mortality, cancer is perceived as a traumatic event or a highly intense stressor among patients that is capable of inducing posttraumatic growth (PTG). PTG is a psychological development that results from a struggle with an intense life stressor or trauma. However, PTG does not develop directly from the traumatic experience. Initially, occurrence of a traumatic event may shatter the assumptive world of a person (a general set of beliefs and assumptions of the person about the surrounding world, which act as guidance for actions and understanding of the causes and reasons of events that happens as well as serves as a sense of meaning and purpose in life). In order to rebuild a new assumptive world after the occurrence of trauma, cognitive rebuilding must take place where there is a search for meaning out of the traumatic experience by incorporating the trauma-related information into reconstruction of the new assumptive world of the person. As a result of these cognitive processes, PTG that develop in a person is a transformative phenomenon which leads to an improved qualitative change of functioning, which surpass the level of functioning before the struggle with the traumatic event or crisis. There may be a curvilinear relationship between the degree of psychological distress and PTG, in which a person with a low level of stress may not be sufficient to trigger PTG compared with someone who experience a trauma, whereas another person with very high degree of trauma may be too overwhelmed to trigger development of PTG (Tedeschi and Calhoun, 1996, 2004; Calhoun et al., 2000).

Posttraumatic growth has been reported in HNC patients (Ho et al., 2011; Leong Abdullah et al., 2015). Higher degree of PTG is associated with greater appreciation of life, improved relationship with others, elevated personal strength, higher spiritual development, and/or increase possibilities in life among cancer patients (Calhoun and Tedeschi, 2006). In addition, PTG is also positively correlated with health-related quality of life (Tomich and Helgeson, 2012; Casellas-Grau et al., 2017) and long-term cancer survivors also reported to have moderate to high level of PTG (Liu et al., 2021). PTG is an important positive psychological change that merits further investigation among HNC patients since it inversely correlates with depression and psychological distress (Shand et al., 2015; Casellas-Grau et al., 2017). However, most of the studies of PTG in cancer patients were cross-sectional in design and are unable to determine how PTG and associated psychological complications varied over time. Interestingly, in a prospective study which investigated the relationship between PTG and depressive symptoms in breast cancer patients across two timelines (between the time of diagnosis and 2years later) reported that depressive symptoms present at the time of diagnosis may facilitate development of PTG provided that depressive symptoms were elevated at the initial period after cancer diagnosis (Romeo et al., 2020).

Various predictors of PTG have been identified in studies on different cancer types. Hope is a goal-directed motivational state which enables one to have a positive outlook in life (Snyder et al., 1991). Hope contributes to several positive outcomes in cancer patients. Higher hope is associated with higher degree of positive psychology, such as PTG, optimism, resilience, and psychosocial adjustment in cancer patients (Hou et al., 2010; Ho et al., 2011; Ryu and Yi, 2013). Conversely, greater hope is negatively associated with depression, anxiety, and psychological distress (Lai et al., 2003; Yang et al., 2014). In the context of Malaysian cancer patients, hope is the most significant associated factor which predicts PTG (Leong Abdullah et al., 2019).

Coping is a behavioral and cognitive process used to tolerate, reduce, or manage the experience of a stressful event (Rajandram et al., 2011). One method of classifying coping strategies is to broadly categorize coping into *approach* and *avoidance* categories (Daisuke and Ayumi, 2016). Coping strategies in cancer patients play a pivotal role as higher level of approach coping, such as acceptance and emotional support predicted higher degree of health-related quality of life and lower degree of depression and anxiety. On the other hand, higher level of avoidance coping, such as denial and self-blaming contributed to lower degree of health-related quality of life and higher degree of depression and anxiety (Nipp et al., 2016). PTG studies on various cancer types have reported that approach coping strategies (such as positive reappraisal and acceptance), as well as religious coping, predict PTG among cancer survivors (Shand et al., 2015; Casellas-Grau et al., 2017). Besides, fatalistic attitude (the belief that regardless of any actions performed, events are predestined to happen) is also associated with higher degree of PTG in breast cancer patients (Romeo et al., 2019). Nevertheless, most of these studies focused on breast cancer and used a cross-sectional design.

In the context of HNC cancer survivors, only two studies had examined the relationship between coping strategies and benefit finding (BF), which indicated that approach coping strategies (such as positive reappraisal, active coping, and emotional support) predicted BF (Harrington et al., 2008; Llewellyn et al., 2013). PTG is distinct from BF; the latter is a process of finding positivity in response to an adverse life event in the form of a perceived benefit, whereas the former involves more pervasive cognitive changes following a traumatic event, shattering assumptions about life (Shand et al., 2015). Moreover, a study on PTG and BF that compared rural and non-rural lung cancer survivors reported that rural survivors had higher PTG than non-rural survivors, whereas no difference was found in BF between the two groups, indicating that PTG and BF are indeed two different constructs (Andrykowski et al., 2017). To the best of our knowledge, to date, no longitudinal studies had investigated how coping strategies relate to PTG over time among HNC survivors. This study aimed to evaluate PTG trends and coping over 5–7months among a cohort of HNC patients within the first year after their diagnosis. It determined an association between coping strategies and PTG over time.

## Materials and Methods

### Study Design and Respondents

This longitudinal study was conducted from January 2019 to December 2020. Its participants were HNC patients registered in the otorhinolaryngology and oncology unit of the Advanced Medical and Dental Institute (AMDI), Universiti Sains Malaysia (USM), and the Universiti Kebangsaan Malaysia Medical Centre (UKMMC). These two university hospitals were selected since these centers are two of the major tertiary referral centers for oncology patients in northern and central Peninsular Malaysia. The sample size required for this study was estimated based on the sample size needed to compare the difference between two dependent means using the G*Power 3.1.9.7 calculator. The following parameters were used for the study’s sample size calculation: effect size=0.231 (based on a prospective study on PTG among HNC patients over 6months by Leong Abdullah et al., 2015), type I error (*α*)=0.05, and power (1-*β*)=0.8. Therefore, the estimated sample size required for this study was 195 (with the addition of a 30% dropout rate).

Consecutive sampling was employed to recruit suitable subjects for this study. All HNC patients who had attended clinic and day care sessions at AMDI, USM, and UKMMC were approached by the study’s research assistant, who was not directly involved in this study, and screened for eligibility criteria. The inclusion criteria included patients who: (1) had been diagnosed with HNC and who had obtained a histopathological report within 1year after diagnosis, (2) were experiencing any stage of HNC, and (3) were 18years old and over. Meanwhile, patients were excluded from the study if they: (1) presented with any history of pre-existing psychiatric illnesses (such as psychotic disorders, depressive disorders, anxiety disorders, bipolar mood disorder, obsessive-compulsive disorder, trauma-related disorders, substance or alcohol use disorders, and substance- or alcohol-related disorders; screened by the research team’s psychiatrist using the *Diagnostic and Statistical Manual for Mental Disorders, 5th Edition*; American Psychiatric Association, 2013), (2) had a history of pre-existing medical illnesses, or (3) were physically too weak to answer questionnaires. HNC patients who met all of the study’s inclusion criteria and none of the exclusion criteria were invited to participate in the study. Patients who voluntarily agreed to participate were explained the study’s procedures, purposes, participation benefits and risks, and assurance of anonymity, as well as their right to withdraw from the study at any point of time, before signing an informed consent form. This study was approved by the Human Ethics Committee of Universiti Sains Malaysia and the Medical Research Committee of Universiti Kebangsaan Malaysia Medical Centre, and it abided by the regulations of the 1964 *Declaration of Helsinki* and its subsequent amendments.

### Data Collection and Measures

#### Data Collection

Data were collected at two time points, during baseline assessments and follow-up assessments (5–7months after baseline assessments). All respondents were administered a socio-demographic and clinical characteristics questionnaire, the Malay version of the “PTG Inventory-Short Form” (PTGI-SF), and the Malay version of the “Brief Coping Orientation to Problems Experienced Inventory” (Brief COPE) during their baseline assessments. The question about treatment modalities, the Malay version of the PTGI-SF and the Malay version of the Brief COPE were re-administered to respondents during their follow-up assessments.

#### Measures

##### Outcome Measure

The Malay version of the PTGI-SF was administered to all respondents in order to assess PTG levels. The “PTG Inventory” (PTGI) is a self-reported instrument comprising 21 items in five domains (*appreciation of life*, *spiritual growth*, *increased personal strength*, *new possibilities in life*, and *improved relationship with others*). The “PTGI-SF” is a shorter version of the PTGI, comprising 10 items in five similar domains (two items per domain). It can replace the PTGI without any loss of information. Each item is scored on a Likert scale, ranging from 0 (*I did not experience this change*) to 5 (*I experienced this change to a great degree*). Thus, the PTGI-SF’s total scores range from 0 to 50. The higher the total score, the greater the degree of PTG a respondent has experienced. The PTGI-SF exhibits good psychometric properties and offers the advantage of shorter administration time than the PTGI (Cann et al., 2010). The Malay version of the PTGI-SF was validated with Malaysian cancer patients, and it demonstrated good internal consistency with Cronbach’s *α*=0.89 (Leong Abdullah et al., 2017).

##### Explanatory Variables

The Malay version of the Brief COPE was administered to respondents in order to measure the coping strategies used by HNC patients in response to cancer as a stressful event. The Brief COPE is a self-reported instrument and a shorter version of the 60-item COPE scale. It is often used in healthcare settings to assess how patients with a life-threatening medical illness cope with a stressful condition, including cancer (Hagan et al., 2017). The Brief COPE comprises 28 items in 14 subscales, such as *positive reframing*, *active coping*, *self-distraction*, *denial*, *substance use*, *use of emotional support*, *behavioral disengagement*, *venting*, *planning*, *humor*, *acceptance*, *religious coping*, *self-blame*, and *use of instrumental support*. Each item is scored on a Likert scale, ranging from 1 to 4. Thus, the total scores for each subscale range from 2 to 8. The Brief COPE subscales can be further categorized into two broad coping styles: *avoidant coping* (comprising *self-distraction*, *denial*, *substance use*, *behavioral disengagement*, *self-blame*, and *venting*) and *approach coping* (comprising *positive reframing*, *active coping*, *use of emotional support*, *planning*, *acceptance*, and *use of instrumental support*). The Brief COPE exhibits good psychometric properties (Carver, 1997). It was validated with the Malaysian cancer population, and its subscales’ internal consistency ranged from Cronbach’s *α* of 0.51–0.99 (Yusoff et al., 2009).

##### Confounding Socio-Demographic and Clinical Characteristics

The study’s data collected during participants’ baseline and follow-up assessments on socio-demographic and clinical characteristics included *gender*, *age*, *religion*, *time since diagnosis*, *stage of cancer*, and *cancer treatment received*. The clinical data’s reliability was confirmed through access to all respondents’ case notes. The response options for *gender* were recorded as *male* or *female*. The choices for age were reported as *18–25*, *26–45*, *45–60years*, or *more than 60years*. Responses to *religion* were recorded as *Islam*, *Buddhism*, *Hinduism*, or *Christianity*. The options for *time since diagnosis* were reported as *less than 6* or *6–12months*. The responses to *stage of cancer* were recorded as *stage 1*, *stage 2*, *stage 3*, or *stage 4*. The responses to *cancer treatment received* were reported as: *no treatment received*; *surgery only*; *chemotherapy only*; *surgery and chemotherapy*; *surgery and radiotherapy*; *chemotherapy and radiotherapy*; or *surgery, chemotherapy, and radiotherapy*.

### Statistical Analysis

All data were analyzed using the Statistical Package for Social Sciences, version 26 (SPSS 26; SPSS Inc., Chicago, Illinois, United States). Descriptive statistics for socio-demographic and clinical characteristics, as well as the total PTGI-SF subscales and Brief COPE scores, were reported. All categorical variables (*gender*, *age*, *religion*, *time since diagnosis*, *stage of cancer*, and *cancer treatment received*) during baseline and follow-up assessments were reported by frequency and percentage. Meanwhile, all the continuous variables (total PTGI-SF and the Brief COPE subscales’ score) were reported as means and standard deviations. The difference in total PTGI-SF and Brief COPE subscale scores between baseline and follow-up assessments were evaluated using a paired *t*-test.

The associations between socio-demographic and clinical variables, and the Brief COPE subscales (independent variables) and total PTGI-SF scores (dependent variables) across baseline and follow-up assessments, were measured using the repeated-measure, mixed-effect model. A random-intercept model was employed since the study’s various measures were not assessed on a pre-determined schedule. The random-intercept model fits a separate intercept and regression line for each respondent, allowing intercepts to vary. The scores for the dependent variables for each observation were, therefore, predicted by the intercepts, which varied across groups. Thus, this approach allowed the effects of all independent variables upon the dependent variables to be evaluated at any point of time between the baseline and follow-up assessments. The statistical significance was *p*<0.05, and all *p* values were two-sided.

## Results

### Respondent Characteristics

Initially, 225 respondents were enrolled in the study. However, only 200 respondents completed the follow-up assessment, while 25 respondents dropped out due to various reasons (such as death, incomplete data, and refusal to participate in follow-up assessment). Thus, the study’s response rate was 89%. All respondents’ socio-demographic and clinical characteristics are summarized in Table 1. Slightly more than half of our respondents were male and in the 45–60-year age group. The majority of our respondents were Muslim. Slightly more than half of our respondents had been diagnosed with HNC less than 6months prior to the study and were experiencing a more advanced stage of cancer (stages 3 or 4). The most common cancer treatment modalities received among participants during their baseline assessments were chemotherapy and radiotherapy, which remained the most common modalities during follow-up assessments.

**Table 1:** Socio-demographic and clinical characteristics of the respondents.

Variables	Frequency (*n*)	Percentage (%)
**Gender**
Male	109	54.5
Female	91	45.5
**Age**		
18–25years	7	3.5
26–45years	48	24.0
45–60years	104	52.0
> 60years	41	20.5
**Religion**
Islam	149	74.5
Buddhism	32	16.0
Hinduism	13	6.5
Christianity	6	3.0
**Time since diagnosis**
< 6months 6–12months	113 87	56.5 43.5
**Stage of cancer:**
Stage 1	43	21.5
Stage 2	54	27.0
Stage 3	65	32.5
Stage 4	38	19.0
**Treatment modalities received (baseline)**
No treatment	25	12.5
Surgery only	24	12.0
Chemotherapy only	38	19.0
Surgery and chemotherapy	13	6.5
Surgery and radiotherapy	25	12.5
Chemotherapy and radiotherapy	52	26.0
Chemotherapy, radiotherapy, and surgery	23	11.5
**Treatment modalities received (follow up)**
Surgery only	7	3.5
Chemotherapy only	28	14.0
Surgery and chemotherapy	23	11.5
Surgery and radiotherapy	24	12.0
Chemotherapy and radiotherapy	73	36.5
Chemotherapy, radiotherapy, and surgery	45	22.5

### Trend of Total PTGI-SF and Brief COPE Subscales Scores Across Baseline and Follow-Up Assessments

The total mean PTGI-SF score significantly increased between the baseline and follow-up assessments (mean baseline score=33.7, *SD*=11.5; mean follow-up score=39.5, *SD*=9.3; *p*<0.001). For the Brief COPE subscales scores, the mean score for *active coping* (*p*=0.001), *planning* (*p*=0.002), *positive reframing* (*p*<0.001), *acceptance* (*p*=0.019), *religious coping* (*p*=0.010), *emotional support* (*p*=0.001), *instrumental support* (*p*=0.001), and *self-distraction* (*p*=0.024) significantly increased between the baseline and follow-up assessments. Additionally, religious coping (*p*=0.010) also significantly increased from baseline to follow-up. In contrast, the mean score for *denial* (*p*<0.001) significantly decreased between baseline and follow-up assessments. The mean scores for the total PTGI-SF and Brief COPE subscales during baseline and follow-up assessments, and the *value of p* of the paired t-test between baseline and follow-up, are presented in Table 2.

**Table 2:** Mean total Posttraumatic Growth Inventory-Short Form (PTGI-SF) and Brief Coping Orientation to Problems Experienced Inventory (Brief COPE) domain scores during baseline and follow up assessment among the respondents.

Variables	Baseline assessment		Follow up assessment		Value of *p* for paired *t* test
	Mean	*SD*	Mean	*SD*	
Total PTGI-SF	33.7	11.5	39.5	9.3	< 0.001^*^
**Brief COPE domains:**
Active coping	6.34	1.39	6.81	1.31	0.001^*^
Planning	6.01	1.57	6.42	1.33	0.002^*^
Positive reframing	6.72	1.37	7.19	1.24	< 0.001^*^
Acceptance	6.74	1.32	7.01	1.08	0.019^*^
Humor	3.24	1.64	3.41	2.00	0.338
Religious coping	7.17	1.35	7.48	0.99	0.010^*^
Emotional support	6.62	1.50	7.07	1.23	0.001^*^
Instrumental support	6.43	1.49	6.87	1.27	0.001^*^
Self-distraction	5.97	1.63	6.29	1.29	0.024^*^
Denial	3.66	1.69	3.04	1.47	< 0.001^*^
Venting	3.99	1.41	4.03	1.32	0.729
Substance use	2.15	0.78	2.04	0.28	0.069
Behavioral disengagement	2.71	1.28	2.54	1.15	0.151
Self-blame	2.87	1.35	2.82	1.13	0.635

* Statistical significance at *p*<0.05.

### Socio-Demographic and Clinical Variables and the Brief Cope Subscales’ Effects on Respondents’ Total PTGI-SF Between Baseline and Follow-Up Assessments

The random-intercept models between the socio-demographic and clinical characteristics, and between the Brief COPE domain scores (independent variables) and total PTGI-SF scores (dependent variable), are summarized in Table 3. The study’s repeated-measure, mixed-effect model revealed that only three Brief COPE subscales significantly predicted respondents’ total PTGI-SF scores between their baseline and follow-up assessments after controlling for relevant socio-demographic and clinical factors. A higher degree of coping by *planning* (estimate=1.256, 95% CI=0.378–2.134, SE=0.447, *t*=2.811, *p*=0.005) and a higher degree of *coping by acceptance* (estimate=1.162, 95% CI=0.188–2.136, SE=0.495, *t*=2.346, *p*=0.019) significantly predicted higher PTG. Meanwhile, a higher degree of *coping by denial* (estimate=−1.078, 95% CI=−1.793 to −0.364, SE=0.363, *t*=−2.966, *p*=0.003) significantly contributed to lower PTG.

**Table 3:** The random intercept model between the socio-demographic and clinical characteristics, and the Brief COPE domain scores (independent variables), and total PTGI-SF scores (dependent variable).

Variables	Estimate (95% CI)	*SE*	*t*	Value of *p*
**Gender**				
Female	Reference			
Male	−2.975 (−5.201 to −0.749)	1.129	−2.635	0.009^*^
**Age**				
18–40years	Reference			
41–60years	4.369 (−0.871 to 9.610)	2.658	1.644	0.102
> 60years	6.479 (0.860–12.098)	2.850	2.273	0.024^*^
**Religion**				
Christianity	Reference			
Islam	2.097 (−3.908 to 8.102)	3.047	0.688	0.492
Buddhism	4.234 (−2.210 to 10.679)	3.269	1.295	0.197
Hinduism	1.782 (−5.447 to 9.010)	3.667	0.486	0.628
**Time since diagnosis**				
< 6months	Reference			
6months to 12months	0.647 (−1.632 to 2.926)	1.156	0.559	0.577
**Stage of cancer**				
Stage 1	Reference			
Stage 2	2.677 (−0.410 to 5.764)	1.566	1.710	0.089
Stage 3	1.986 (−1.147 to 5.118)	1.589	1.250	0.213
Stage 4	1.790 (−1.654 to 5.233)	1.747	1.025	0.307
**Treatment modalities received**				
No treatment	Reference			
Surgery only	1.598 (−3.641 to 6.837)	2.665	0.600	0.549
Chemotherapy only	6.015 (1.262 to 10.768)	2.418	2.488	0.013^*^
Surgery and chemotherapy	5.503 (0.194 to 10.812)	2.700	2.038	0.042^*^
Surgery and radiotherapy	1.134 (−3.934 to 6.201)	2.578	0.440	0.660
Chemotherapy and radiotherapy	5.174 (0.866–9.483)	2.191	2.362	0.019^*^
Chemotherapy, radiotherapy, and surgery	3.360 (−1.385 to 8.104)	2.413	1.392	0.165
**The Brief COPE domains**				
Active coping	−0.159 (−0.985 to 0.667)	0.420	−0.378	0.705
Planning	1.256 (0.378 to 2.134)	0.447	2.811	0.005^*^
Positive reframing	0.608 (−0.484 to 1.699)	0.555	1.094	0.275
Acceptance	1.162 (0.188 to 2.136)	0.495	2.346	0.019^*^
Humor	−0.186 (−0.759 to 0.387)	0.291	−0.638	0.524
Religious coping	0.756 (−0.196 to 1.707)	0.484	1.561	0.119
Emotional support	0.384 (−0.736 to 1.504)	0.570	0.674	0.501
Instrumental support	0.175 (−0.894 to 1.245)	0.544	0.322	0.748
Self-distraction	−0.021 (−0.816 to 0.774)	0.404	−0.052	0.958
Denial	−1.078 (−1.793 to −0.364)	0.363	−2.966	0.003^*^
Venting	0.010 (−0.823 to 0.843)	0.424	0.023	0.982
Substance use	0.804 (−1.117 to 2.725)	0.977	0.823	0.411
Behavioral disengagement	0.712 (−0.299 to 1.724)	0.515	1.385	0.167
Self-blame	−0.287 (−1.219 to 0.644)	0.474	−0.607	0.544

* Statistical significance at *p*<0.05.

As for socio-demographic characteristics, male HNC respondents registered a significantly lower degree of PTG compared with female HNC respondents across time (estimate=−2.975, 95% CI=−5.201 to −0.749, SE=1.129, *t*=−2.635, *p*=0.009), whereas those aged above 60years old exhibited a significantly higher degree of PTG compared with those aged 18–40years old (estimate=6.479, 95% CI=0.860–12.098, SE=2.850, *t*=2.273, *p*=0.024). Treatment modalities received was the only clinical characteristic associated with PTG across time, whereby those who received chemotherapy only (estimate=6.015, 95% CI=1.262–10.768, SE=2.418, *t*=2.488, *p*=0.013), surgery and chemotherapy (estimate=5.503, 95% CI=0.194–10.812, SE=2.700, *t*=2.038, *p*=0.042), and chemotherapy and radiotherapy (estimate=5.174, 95% CI=0.866–9.483, SE=2.191, *t*=2.362, *p*=0.019) demonstrated significantly higher degree of PTG compared with those who were treated with surgery only (Table 3).

## Discussion

This study investigated PTG and coping trends across 5–7months among a cohort of HNC patients within the first year after their diagnosis. It determined coping strategies’ effect on PTG over time. We found that PTG and approach coping (active coping, planning, positive reframing, acceptance, emotional support, and instrumental support) increased while avoidant coping (self-distraction and denial) decreased over time (from baseline to follow-up). Moreover, two approach coping styles – namely, planning and acceptance – contributed to higher PTG over time. On the contrary, one avoidant coping style – namely, denial – precipitated lower PTG over time among HNC respondents after controlling for socio-demographic and clinical characteristics.

The increasing PTG trend among HNC patients has been well-documented during their first 18months of cancer diagnosis (Harding, 2018a). Our findings confirmed the PTG trend during patients’ first 18months after diagnosis since our respondents had been diagnosed with cancer with the past 12months of the study, and the time interval between their baseline and follow-up assessments was 5–7months; thus, respondents had been diagnosed up to 18months previously to their follow-up assessments in this study. Further, the trend of coping over time after an initial cancer diagnosis in our findings confirmed the findings of previous studies with cancer patients, in which approach coping – such as acceptance, active coping, positive reframing (through positive cognitive restructuring), and seeking emotional and informational support – and religious coping increased over time, whereas avoidant coping (such as denial and self-distraction) depreciated gradually as acceptance increased (Sajadian et al., 2017). Moreover, another study evaluating coping among HNC survivors also indicated that positive reframing, active coping, religious coping, and seeking emotional support from family and friends are common coping strategies after diagnosis (Jagannathan and Juvva, 2016).

Our findings revealed that approach coping helped increase PTG over time. For instance, a higher degree of acceptance significantly enhanced respondents’ PTG. To the best of our knowledge, to date, our study is the first to confirm the acceptance’s positive effect on PTG among HNC survivors. This positive relationship between acceptance and PTG was further strengthened by similar findings that have been reported among breast cancer survivors, which indicated that acceptance lowers perceived stress and, in turn, enhances PTG over time (Bussell and Naus, 2010). Moreover, longitudinal study in cancer patients also reported that problem-focus coping (coping by direct confrontation of stress to reduce or eliminate it, which is similar to approach coping) and active-adaptive coping predicted higher degree of PTG over time (Scrignaro et al., 2011; Danhauer et al., 2015). This finding supported the theoretical model suggesting that PTG is an adaptive process of cognitively processing a traumatic event, during which approach coping may moderate PTG (Bussell and Naus, 2010). Thus, we propose that acceptance of the traumatic event (cancer diagnosis) is an important initial process (Prati and Pietrantoni, 2009) that allows the cognitive reappraisal of the traumatic (cancer) experience before a successful search for meaning in the trauma can facilitate PTG development among cancer survivors.

Interestingly, a higher degree of planning for ways to cope with and manage the stressful event of cancer also tends to enhance PTG over time, as our study has highlighted. Greater hope has been reported to predict higher levels of PTG among HNC patients (Ho et al., 2011). Hope is associated with positive beliefs and attitudes about posttraumatic worldviews in relation to the self, others, and the surrounding world. Greater hope facilitates cognitive reappraisal, allowing HNC patients to rethink the discrepancies between their pre-trauma and post-trauma worldviews, incorporate new trauma-related information, and form a new posttraumatic worldview of the self, others, and the surrounding world *via* accommodation, therefore facilitating the PTG development among cancer survivors (Leong Abdullah et al., 2019). The planning coping strategy and pathway component of hope share similarities (the perceived ability to generate ways and paths to achieve a goal-set). A previous study of HNC survivors also identified that the pathway component of hope was particularly associated with increased PTG (Ho et al., 2011). Our study’s HNC survivors may have exhibited a high level of hope’s pathway component, increasing their tendency to plan for ways to cope with and manage the stressful event of cancer and, in turn, facilitating PTG development.

Unsurprisingly, a higher degree of coping by denial lowered the degree of PTG among HNC survivors over time. There is a growing body of literature indicating that avoidant coping contributes to lower degree of PTG among cancer patients (Kroemeke et al., 2017; Li et al., 2019). Denial about having cancer and cancer’s negative consequences may have occurred at a higher degree immediately after cancer diagnoses. Denial or self-deception may have allowed patients to shift their perceptions from losses to benefits, thereby consolidating and maintaining an assumptive worldview of the self, others, and the surrounding world in response to trauma, facilitating the development of PTG’s illusory aspect to counteract the emotional distress stemming from their traumatic events. This cognitive avoidance coping disrupts the cognitive reappraisal of cancer as a traumatic event and encourages the avoidance of a search for meaning in the traumatic event. Consequently, though denial may offer short-term relief of emotional distress, it is maladaptive over the long term, preventing the development of the PTG’s constructive aspect (Zoellner and Maercker, 2006; Ochoa Arnedo et al., 2019). This finding may explain the inverse relationship between denial and PTG among this study’s respondents.

As for the association between socio-demographic characteristics and PTG in cancer patients, gender was reported to be associated with PTG, wherein females tend to exhibit higher degree of PTG than males (Shand et al., 2015; Sharp et al., 2018; Leong Abdullah et al., 2019). Similar finding was also reported among HNC patients (Holtmaat et al., 2017) and the finding of our study further strengthened the association between female cancer patients and higher degree of PTG. It has been suggested that females tend to engage in cognitive reprocessing of the trauma-related experience, initiate search for meaning out of the traumatic experience and thus, increasing the likelihood of developing PTG as compared to males (Leong Abdullah et al., 2019). Besides gender, this study also revealed that older HNC patients (above 60years old) had greater degree of PTG compared with younger patients. This contradicts the finding of a longitudinal study in breast cancer patients which documented patients of younger age group had higher degree of PTG (Danhauer et al., 2015). But in the context of HNC patients, no published studies have found an impact of age on PTG (Harding, 2018b). Hence, the relationship between age and PTG in cancer patients remains inconsistent.

Several studies have investigated the association between cancer treatment modalities (chemotherapy, radiotherapy, and surgery) and PTG among cancer patients (Tanyi et al., 2017). Among the modalities of treatment, chemotherapy has been shown to be a significant predictor of higher PTG among cancer patients across time (Danhauer et al., 2015). Our findings were consistent with that of other study as HNC patients who received chemotherapy, either as monotherapy or in combination with other treatment modalities, experienced higher PTG over time compared with those who were on surgery alone. Chemotherapy may greatly disrupt the daily living and induced significant degree of stress among the HNC patients due to the adverse effects associated with this treatment modality. This may initiate the adaptive processes to search for meaning out of their stressful experience and enable positive psychological changes such as PTG to develop among the HNC respondents in our study (Danhauer et al., 2015). Regarding other socio-demographic and clinical characteristics, such as religion, time since diagnosis, and stage of cancer, they were no association between them and PTG in cancer patients as indicated by other longitudinal studies (Danhauer et al., 2013, 2015; Leong Abdullah et al., 2015).

Our findings should be interpreted alongside a consideration of a few limitations. First, respondents’ socio-demographic characteristics in this study may not sufficiently represent Malaysia’s HNC patient population. This limitation affects our findings’ generalizability. Finally, although we proposed that the effect of coping by planning on a higher degree of PTG may be mediated by hope’s pathway component, we did not assess hope in this study. Therefore, we recommend that future longitudinal studies assess the relationship between coping, hope, and PTG.

Notwithstanding these limitations, this study is the first longitudinal study in HNC survivors to evaluate how coping styles vary over time, as well as coping styles’ effect on PTG over time. Our findings suggest that treating clinicians must incorporate psychosocial interventions that effectively increase acceptance and reduce denial into their treatment regimes for HNC patients, such as acceptance and commitment therapy (ACT; Dindo et al., 2017; Zhao et al., 2021). ACT facilitates the development of greater psychological flexibility by increasing adaptive coping through acceptance, cognitive diffusion, mindfulness, and perspective-taking exercises, and it supports cancer survivors’ aligning their behaviors with their personal values (Johns et al., 2019). Therefore, ACT may help enhance PTG among HNC survivors by increasing acceptance and reducing denial.

As we have shown, this study was the first longitudinal study to show that approach coping (such as active coping, planning, positive reframing, acceptance, emotional support, and instrumental support) increased while avoidant coping (self-distraction and denial) decreased over time among a cohort of HNC survivors within the first year of their diagnoses. Two approach coping strategies enhanced PTG over time – namely, acceptance and planning. In contrast, the only avoidant coping that reduced PTG over time was denial. Based on our findings, we have suggested that future research investigate ACT’s efficacy in enhancing PTG among HNC survivors. If ACT’s efficacy on PTG is documented, ACT’s inclusion in HNC patients’ treatment regime may be pivotal.

## Data Availability Statement

The original contributions presented in the study are included in the article, further inquiries can be directed to the corresponding author.

## Ethics Statement

The studies involving human participants were reviewed and approved by the Human Research Ethics Committee of USM, Division of Research & Innovation (R&I), USM Health Campus, Kubang Kerian, Kelantan, Malaysia, and the Medical Research Committee of the Faculty of Medicine, Universiti Kebangsaan Malaysia, Cheras, Kuala Lumpur, Malaysia. The patients/participants provided their written informed consent to participate in this study.

## Author Contributions

ML and NN conceptualized and designed the study and involved in data and statistical analysis. ML, NN, NAH, NA, RR, RM, MM, and HZ involved in data collection. ML wrote the first draft of the manuscript. All authors contributed to the article and approved the submitted version.

## Funding

This work was supported by the Short Term Grant of Universiti Sains Malaysia (Grant number: 304/CIPPT/6315236; author ML). The funder has no role in the conceptualization of the review, literature review, writing of the manuscript, and decision on submission of the manuscript for publication. There was no fund received for open access publication fees.

## Conflict of Interest

The authors declare that the research was conducted in the absence of any commercial or financial relationships that could be construed as a potential conflict of interest.

## Publisher’s Note

All claims expressed in this article are solely those of the authors and do not necessarily represent those of their affiliated organizations, or those of the publisher, the editors and the reviewers. Any product that may be evaluated in this article, or claim that may be made by its manufacturer, is not guaranteed or endorsed by the publisher.
